# Metabolic syndrome predictors of brain gray matter volume in an age-stratified community sample of 776 Mexican- American adults: Results from the genetics of brain structure image archive

**DOI:** 10.3389/fnagi.2022.999288

**Published:** 2022-09-20

**Authors:** Eithan Kotkowski, Larry R. Price, Ralph A. DeFronzo, Crystal G. Franklin, Maximino Salazar, Amy S. Garrett, Mary Woolsey, John Blangero, Ravindranath Duggirala, David C. Glahn, Peter T. Fox

**Affiliations:** ^1^Research Imaging Institute, University of Texas Health Science Center at San Antonio, San Antonio, TX, United States; ^2^Departments of Mathematics and Education, Texas State University, San Marcos, TX, United States; ^3^Diabetes Research Unit and Diabetes Division, Texas Diabetes Institute, University of Texas Health Science Center at San Antonio, San Antonio, TX, United States; ^4^Genomics Computing Center, South Texas Diabetes and Obesity Institute, University of Texas Rio Grande Valley, Brownsville, TX, United States; ^5^Department of Psychiatry, Yale University School of Medicine, New Haven, CT, United States; ^6^Olin Neuropsychiatry Research Center, Institute of Living, Hartford Hospital, Hartford, CT, United States; ^7^South Texas Veterans Health Care System, San Antonio, TX, United States

**Keywords:** metabolic syndrome, insulin resistance, central obesity, waist circumference, magnetic resonance imaging (MRI), voxel-based morphometry (VBM), posterior cerebellum, cerebellar cognitive affective syndrome (CCAS)

## Abstract

**Introduction:**

This project aimed to investigate the association between biometric components of metabolic syndrome (MetS) with gray matter volume (GMV) obtained with magnetic resonance imaging (MRI) from a large cohort of community-based adults (*n* = 776) subdivided by age and sex and employing brain regions of interest defined previously as the “Neural Signature of MetS” (NS-MetS).

**Methods:**

Lipid profiles, biometrics, and regional brain GMV were obtained from the Genetics of Brain Structure (GOBS) image archive. Participants underwent T1-weighted MR imaging. MetS components (waist circumference, fasting plasma glucose, triglycerides, HDL cholesterol, and blood pressure) were defined using the National Cholesterol Education Program Adult Treatment Panel III. Subjects were grouped by age: early adult (18–25 years), young adult (26–45 years), and middle-aged adult (46–65 years). Linear regression modeling was used to investigate associations between MetS components and GMV in five brain regions comprising the NS-MetS: cerebellum, brainstem, orbitofrontal cortex, right insular/limbic cluster and caudate.

**Results:**

In both men and women of each age group, waist circumference was the single component most strongly correlated with decreased GMV across all NS-MetS regions. The brain region most strongly correlated to all MetS components was the posterior cerebellum.

**Conclusion:**

The posterior cerebellum emerged as the region most significantly associated with MetS individual components, as the only region to show decreased GMV in young adults, and the region with the greatest variance between men and women. We propose that future studies investigating neurological effects of MetS and its comorbidities—namely diabetes and obesity—should consider the NS-MetS and the differential effects of age and sex.

## Introduction

Metabolic syndrome (MetS), also known as the insulin resistance syndrome, systemic metabolic dysfunction, or syndrome X (Reaven, 1988), is a cluster of clinical risk factors for cardiovascular disease (CVD) and type 2 diabetes mellitus (T2DM). These risk factors include central obesity [increased waist circumference (WC)], elevated fasting plasma glucose (FPG), triglycerides (TG), blood pressure (BP), and reduced high density lipoprotein (HDL) cholesterol (Grundy et al., 2005). MetS and its associated cardiometabolic diseases—CVD, T2DM, and obesity—are also known to be significantly associated with cognitive dysfunction (Geijselaers et al., 2014; Gonzalez et al., 2015) and neurodegenerative diseases such as Alzheimer’s and vascular dementia (Vanhanen et al., 2006; Lee and Mattson, 2014; Morris et al., 2014). Indeed, there are measurable significant negative correlations seen between both the number and severity of MetS components when measured against cognition metrics, specifically for tests of executive function (Yates et al., 2012), processing speed (Reijmer et al., 2012), reward perception (Cornier et al., 2010; Cameron et al., 2017), and affect regulation (Hu et al., 2015; Wolf et al., 2016).

Despite numerous studies examining the cognitive effects of MetS, neuroimaging studies aimed at identifying gray matter neuroanatomical correlates of MetS are sparse. Existing neuroimaging literature has focused primarily on white matter integrity and hyperintensities (Van Bloemendaal et al., 2016), structures associated with processing speed (Kochunov et al., 2016), but not with behavior or executive function (Lansley et al., 2013). Furthermore, there is a sizeable neuroimaging literature investigating correlations between obesity markers such as body mass index (BMI), waist circumference and gray matter volume (Kurth et al., 2013; Janowitz et al., 2015). Many such studies have often excluded individuals possessing MetS components as confounders, in effect eliminating subjects with a higher disease burden who are more likely to experience neurovascular events and cognitive decline. Furthermore, most large cohort studies looking at diabetes and obesity have primarily been conducted in older (>60 years) individuals, Caucasian and East Asian ethnic cohorts, and have often employed relatively small sample sizes (30–50 subjects) (Moulton et al., 2015; Wu et al., 2017).

Age and sex are important sources of variance in any analysis involving MetS and neuroimaging. It is widely understood that sex hormones significantly influence fat metabolism and adipocyte distribution, an influence that also changes with age. Indeed, metabolic studies have shown that men tend to exhibit higher levels of triglycerides and blood pressure, but lower age-adjusted levels of waist circumference and HDL cholesterol as compared to women (Ervin, 2009). Effects of MetS also differ between men and women physiologically (Regitz-Zagrosek et al., 2006) and cognitively (Laudisio et al., 2008). In one example, Cavalieri et al. (2010) found that men with increasing number of MetS components performed worse on memory and executive function tests than women. Another more recent study showed that higher waist circumference and mental health symptoms had a stronger association with dementia in women than in men (Gong et al., 2021). For this reason, investigating the effects of MetS on the brain using separate sex-stratified cohorts is important. Assessing similarities and differences between the sexes can be accomplished using regression analyses that adjust for the effects of age and sex. However, we believe that stratifying groups as independent analyses can be more helpful in providing valuable insight for clinicians to be mindful of the differential effects of MetS and its neurocognitive underpinnings on men vs. women.

Age is also an important risk factor for MetS. One large epidemiological study of the United States reported that 18.3% of individuals aged 20–39 years met the International Diabetes Federation’s criteria for MetS with the prevalence rising sharply with age. Indeed, 46.7% of those older than 60 years were identified as meeting the diagnostic criteria for MetS. Importantly, the age of any individual with MetS can be confounded by the length of time they have met the diagnostic criteria, and whether meeting such criteria occurred earlier vs. later in life. The prevalence of MetS is also higher among minority populations, with Hispanic-Americans and Native Americans most widely affected (Aguilar et al., 2015). As with sex, linear regression modeling can adjust for the effects of age. In fact, it is often considered the *sine qua non* of such analyses. However, independent age-stratified analyses may shed light on whether there are neuroanatomical differences seen in cross sections of early vs. young vs. middle-aged adults, and whether age is still a significant co-factor within the stratified age-groups.

**FIGURE 1:**
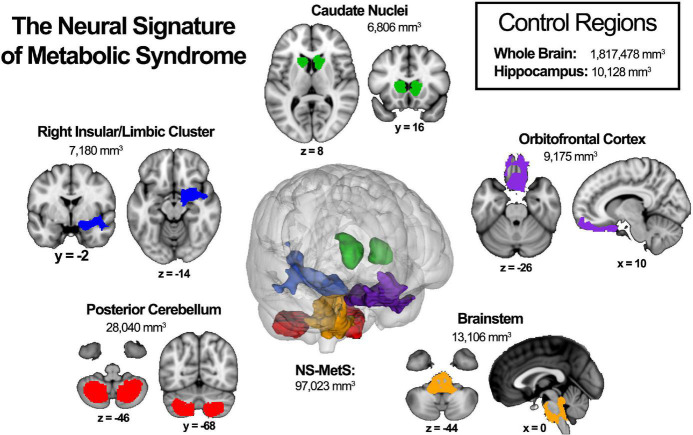
The neural signature of metabolic syndrome (NS-MetS) as defined by Kotkowski et al. (2019). Each indicated region represents one of the five main clusters that comprise the NS-MetS. Control regions include gray matter densities from the whole brain and hippocampal, neither of which are part of the NS-MetS.

Previously, our lab characterized the neural signature of MetS (NS-MetS) using a large age- and sex-matched cohort (*n* = 208; 37.3 ± 13.2 years, 56.7% women) of Mexican-American participants, 104 meeting the International Diabetes Federation criteria for MetS and 104 healthy controls that did not meet any criteria. Those with MetS were observed to have lower gray matter volume in specific brain regions as compared to their age- and sex-matched metabolically healthy controls. These five chief brain regions included the posterior cerebellum, brainstem, orbitofrontal cortex, right insula/limbic structures, and caudate nuclei (Kotkowski et al., 2019; Figure 1). Interestingly, decreased GMV within the hippocampus was conspicuously absent from the findings. This was surprising because the hippocampus is the structure whose degeneration is touted as the primary feature in the pathophysiology of Alzheimer’s disease. It is also often the most often alleged culprit in much of the diabetes and obesity-related dementia literature (Biessels and Reagan, 2015; Alford et al., 2018).

For the present study, we sought to identify the extent to which brain regions making up the neural signature of MetS are related to the biometric components that define MetS. This will allow us to specify the metabolic underpinnings associated with reduced gray matter volume in individuals with MetS and identify how they differ between age-groups and by sex. We therefore hypothesized that gray matter volume will exhibit an inverse linear relationship with each MetS components across our community-based population (with the exception of HDL cholesterol), implying that the relationship between NS-MetS GMVs in these regions and individual MetS components lie on a continuum. We also hypothesized that sex and age would show variations in the relationship between gray matter and MetS, crucial factors necessary for the design of future studies and treatment plans. Moreover, we included whole brain gray matter and hippocampal gray matter as regions of interest in our analyses due to their high research interest with the hypothesis that they will show weaker correlations with MetS components than NS-MetS regions.

## Materials and methods

### Participants

The Genetics of Brain Structure (GOBS) dataset is a cross-sectional imaging archive aimed at localizing, identifying, and characterizing genes and quantitative trait loci associated with variations in brain structure and function. To minimize selection bias, participants were drawn from extended pedigrees of Mexican American families recruited at random from the San Antonio, Texas community. Demographic and data acquisition details have been previously described (Winkler et al., 2010; Curran et al., 2013; Kotkowski et al., 2019). Briefly, 1,911 individuals were recruited in three data acquisition blocks over the course of 15 years. The sample in the current manuscript was selected from the GOBS cohort based on strict inclusion criteria. Participants must have completed structural MRI scans and acquired all MetS component metrics: fasting plasma glucose, lipid panel, blood pressure, and waist circumference. A notable limitation of this cohort is its lack of more precise glucose measures in the form of hemoglobin A1c.

Of the 1,911 participants in the GOBS archive, 519 were excluded due to lack of or inadequate, incomplete, or absent T1-weighted MRI scans. An additional 557 participants from the first phase of acquisition were excluded due to a lack relevant biometric, blood chemistry, and lipid data relevant to MetS. Thirty Five subsequent participants with a history of stroke, neurosurgery, dementia, or substance use disorder also were excluded from the analysis. Finally, 24 individuals 66-years and older, were excluded because the cohort size, which would have been termed “older adults,” was not large enough to produce statistically significant data. This left a total of 776 individuals in the final sample. These individuals were further sub-divided arbitrarily into three age-groups of roughly similar size and by sex as follows: early adult (18–25 years) men (*n* = 137) and women (*n* = 148), young adult (26–45 years) men (*n* = 137) and women (*n* = 183), and middle-age (46–65 years) men (*n* = 67) and women (*n* = 103).

### Magnetic resonance imaging

Scans were obtained on a 3T Siemens Tim Trio MRI scanner at the University of Texas Health Science Center at San Antonio’s Research Imaging Institute with the aid of an 8-element high-resolution phase array head coil. A total of seven high quality T1-weighted 3D structural images were collected per participant *via* a TurboFLASH sequence with an adiabatic inversion recovery pulse (TE = 3.04 ms, TR = 2,000 ms, TI = 795 ms, flip angle = 8°, NEX = 6; FOV = 200 mm) optimized to achieve a gray/white contrast of approximately 25% with signal to noise ratio of 25. Each image contained 0.8 mm^3^ isotropic voxels and covered the entire brain and cerebellum. Scan time per participant for the anatomical T1-weighted 3D scan totaled 60 min. Afterward, each of the seven MPRAGE T1-weighted images was motion corrected using a retrospective motion-correction protocol and averaged to generate a single high-resolution anatomical image per participant. This method of acquisition produces high quality images because it breaks down a long acquisition time into several shorter acquisitions that are then corrected using inter-scan motion. This is done by registering images to the third image and forming a single image that is both averaged and motion-corrected, thus improving contrast-to-noise ratio and boundary detail (Kochunov et al., 2006).

### Gray matter volume

Brain volume was calculated using the FMRIB Software Library (FSL) voxel-based morphometry (VBM) pipeline (Good et al., 2001; Smith et al., 2004). We used Freesurfer for initial processing with the *autorecon1* command that corrects head motion, normalizes intensities, and skull-strips the brain for further processing (Fischl, 2012). Skull-stripped brains were then processed with FSL-VBM (Douaud et al., 2007). The next step entailed segmenting gray-matter by also eliminating the influence of white matter and cerebrospinal fluid and performing a non-linear registration to the Montreal Neurological Institute (MNI)-152 1 × 1 × 1 standardized brain space (Andersson et al., 2007). A study-specific template was then created to which all images were subsequently registered. Images were then modulated to correct for expansion (or contraction) resulting from the spatial transformation from native space to the standard MNI-125 template space. Finally, gray matter images were smoothed with an isotropic Gaussian kernel (sigma = 3 mm).

Once processed, normalized gray matter volumes were extracted from each individual brain by applying region of interest (ROI) masks for the entire neural signature of metabolic syndrome (NS-MetS), and each of the five largest NS-MetS sub-regions, including: the posterior cerebellum, brainstem, orbitofrontal cortex, right insular/limbic cluster and caudate (Figure 1). Additionally, we also measured hippocampal and whole brain ROIs, due to the high interest in these regions in the MetS, diabetes, and obesity literature. Normalized values of gray matter density were calculated by taking the total gray matter signal from each ROI in each normalized image and dividing it by the total ROI volume for each relevant ROI.

### Group analysis

All group analyses were performed using SPSS software (version 25; SPSS Inc., Cary, Chicago, United States). Two-tailed *p*-values < 0.008 were considered statistically significant after correcting for multiple comparisons between the six groups in our study (early adult men and women, young adult men and women, and middle-aged men and women). Because fasting plasma glucose (FPG) and triglyceride (TG) levels are not normally distributed and have a positive skew, we applied a log-transform to these values to fit the linear regression framework. We then assessed the differences between the three age groups within each sex using a one-way analysis of variance (ANOVA) followed by a separate one-way ANOVA comparing men and women. Finally, we performed two separate (for each sex) age-adjusted partial Pearson’s correlations to probe the association between MetS’s component values, gray matter densities, and other related biometric values of interest (LDL cholesterol, total cholesterol, body mass index, and NCEP-ATP III MetS score).

A total of six univariate linear regression analyses were performed independently within in each of the six age/sex groups. We chose this approach in order to identify the MetS components (independent variables) that would most strongly correlate with GMV within select regions of interest (dependent variables). All MetS variables plus age were entered in a single linear regression step. This method allowed us to correct for each variable’s influence on every other variable and subsequently identify the most significant variable or variables accounting for the observed correlations.

## Results

### Demographics

The demographic and MetS component characteristics of our cohort can be found in Table 1 where the most appreciable between-sex differences (*p*≤ 0.001) were observed in triglycerides (TG), high density lipoprotein (HDL) cholesterol, diastolic blood pressure (BPD), and National Cholesterol Education Program Adult Treatment Panel III (NCEP-ATP III) scores for MetS. Notably, there were no significant differences seen between the sexes with respect to BMI, total cholesterol, low density lipoprotein (LDL) cholesterol, and global assessment of functioning (GAF) scores. There were also no significant differences in the percentage of participants with type 2 diabetes, hypercholesterolemia, hypertriglyceridemia, and hypertension between the sexes or in individuals taking prescribed medications for each of these disorders. As hypothesized, every within-sex and between-age measure was significantly different in men with the exception of HDL cholesterol and Global Assessment of Function (GAF) scores. Similarly, each within-sex and between-age measure was significantly different in women with the exception of GAF scores.

**TABLE 1:** Group demographic data and health characteristics (mean ± SD).

	Men				Women				*P-*value^†^
	Early adult: 18–25 years	Young adult: 26–45 years	Middle-age adult: 46–65 years	*P*-value*	Early adult: 18–25 years	Young adult: 26–45 years	Middle-age adult: 46–65 years	*P*-value	
Sample (*n*)	137	137	67		148	183	103		
Age (years)	20.8 ± 2.2^A^	34.8 ± 6.1^B^	54.9 ± 5.7^C^	<0.001	20.7 ± 2.2^A^	34.7 ± 5.9^B^	53.1 ± 5.3^C^	<0.001	0.229
Education (years)	12.0 ± 1.2^A^	12.1 ± 2.2^A^	11.5 ± 3.0^A^	0.162	12.4 ± 1.8^A^	12.7 ± 2.5^A^	11.6 ± 3.3^B^	0.002	0.027
Waist Circumference (cm)	94.7 ± 17.8^A^	104.2 ± 15.2^B^	108.0 ± 12.1^B^	<0.001	94.0 ± 17.2^A^	100.3 ± 13.4^B^	102.0 ± 12.6^B^	<0.001	0.027
Fasting glucose (mg/dL)	90.7 ± 9.8^A^	105.4 ± 46.7^B^	123.1 ± 51.1^C^	<0.001	88.6 ± 24.4^A^	98.4 ± 40.8^B^	120.7 ± 71.4^C^	<0.001	0.042
Triglycerides (mg/dL)	118.8 ± 75.4^A^	173.2 ± 119.1^B^	199.0 ± 381.2^B^	<0.001	101.7 ± 45.4^A^	137.4 ± 137.9^B^	136.8 ± 73.1^B^	<0.001	0.001
HDL cholesterol (mg/dL)	48.1 ± 12.5	44.8 ± 12.8	47.7 ± 14.9	0.090	50.7 ± 11.8^AB^	50.6 ± 15.6^A^	54.9 ± 15.9^B^	0.037	<0.001
Blood pressure (mmHg)									
Systolic	119.0 ± 12.4^A^	122.7 ± 16.8^A^	128.4 ± 20.6^B^	0.001	113.2 ± 11.5^A^	120.8 ± 17.3^B^	125.2 ± 16.4^B^	<0.001	0.011
Diastolic	72.1 ± 9.9^A^	77.2 ± 13.1^B^	76.6 ± 10.4^B^	0.001	68.7 ± 10.9^A^	74.1 ± 11.5^B^	74.5 ± 9.6^B^	<0.001	0.001
Type 2 diabetes (%)	0.73%^A^	8.76%^B^	28.4%^C^	<0.001	2.70%^A^	11.5%^B^	27.2%^C^	<0.001	0.212
Hypertension (%)	5.11%^A^	21.2%^B^	40.3%^C^	<0.001	3.38%^A^	13.7%^B^	37.9%^C^	<0.001	0.344
Hypertriglyceridemia (%)	0.73%^A^	5.84%^A^	13.4%^B^	0.001	0.00%^A^	3.83%^A^	16.5%^B^	<0.001	0.878
Hypercholesterolemia (%)	2.92%^A^	12.4%^B^	37.3%^C^	<0.001	1.35%^A^	8.74%^A^	26.2%^B^	<0.001	0.181
NCEP-ATP III score	1.8 ± 2.3^A^	4.0 ± 2.8^B^	4.8 ± 3^B^	<0.001	3.0 ± 2.3^A^	4.1 ± 2.7^B^	5.2 ± 2.8^C^	<0.001	0.001
Body mass index (kg/m^2^)	28.3 ± 6.9^A^	31.1 ± 6.2^B^	31.6 + 4.7^B^	<0.001	29 ± 7.5^A^	31.5 ± 6.8^B^	32.6 ± 6.1^B^	<0.001	0.081
Total cholesterol (mg/dL)	171.4 ± 29.8^A^	191.2 ± 40.7^B^	190.9 ± 51.4^B^	<0.001	162.4 ± 29.6^A^	186.0 ± 35.6^B^	195.8 ± 39.0^B^	<0.001	0.303
LDL cholesterol (mg/dL)	100.3 ± 30.5^A^	113.2 ± 37.3^B^	111.2 ± 38.9^AB^	0.007	91.4 ± 27.1^A^	109.6 ± 28.4^B^	114.2 ± 34.0^B^	<0.001	0.187
Composite of psychometric scores (normalized values)	0.54 ± 0.15^A^	0.53 ± 0.16^AB^	0.48 ± 0.16^B^	0.022	0.49 ± 0.15^AB^	0.51 ± 0.14^A^	0.45 ± 0.15^B^	0.002	0.001
Global assessment of functioning (scale 1–100)	76.5 + 12.1	76.2 ± 12.5	72.2 ± 13.8	0.968	77.3 ± 11.9	78.7 ± 12.3	76.1 ± 12.5	0.206	0.164

Data represented as percent gray matter volume within each given region of interest cluster (mean ± SD).

^*^Results of one-way ANOVA representing age-group differences within each sex.

^†^Results of one-way ANOVA representing between-sex differences.

^A,B,C^Within each row and within each sex group, each letter represents significantly different group means based on Tukey’s *post hoc* testing. All diagnoses based on active treatment for specified condition.

Table 2 lists gray matter densities (GMV) for each region of interest obtained from voxel-based morphometry (VBM) processing arranged by sex- and age-group, previously described. As with Table 1, the results highlight the differences between men and women and differences between age groups. The brain regions whose gray matter densities were significantly different between men and women (*p* ≤ 0.001) included the whole brain, neural signature of metabolic syndrome (NS-MetS) regions as a whole, orbitofrontal cortex, right insular/limbic cluster, the caudate nuclei (*p* = 0.003), and brainstem (*p* = 0.013). Notable between sex difference exceptions included the posterior cerebellum (*p* = 0.254) and hippocampus (*p* = 0.829). Importantly, results did not change when covaried for total brain volume. Interestingly, differences between age-groups and within sex-groups in all brain regions’ GMVs were significantly different (*p* ≤ 0.001) from one another with the notable exception of the hippocampus (*p* = 0.079 for men and *p* = 0.222 for women).

**TABLE 2:** Brain gray matter volume statistics by age, sex, and brain regions.

	Men				Women				*P*-value^†^
	Early adult: 18–25 years	Young adult: 26–45 years	Middle-age adult: 46–65 years	*P*-value*	Early adult: 18–25 years	Young adult: 26–45 years	Middle-age adult: 46–65 years	*P*-value*	
Whole brain	0.50 ± 0.01^A^	0.47 ± 0.02^B^	0.44 ± 0.01^C^	<0.001	0.51 ± 0.01^A^	0.49 ± 0.02^B^	0.46 ± 0.02^C^	<0.001	<0.001
Neural signature of metabolic syndrome	0.56 ± 0.06^A^	0.51 ± 0.07^B^	0.47 ± 0.08^C^	<0.001	0.57 ± 0.05^A^	0.54 ± 0.06^B^	0.50 ± 0.06^C^	<0.001	0.001
Posterior cerebellum	0.73 ± 0.15^A^	0.64 ± 0.16^B^	0.56 ± 0.17^C^	<0.001	0.74 ± 0.10^A^	0.66 ± 0.12^B^	0.59 ± 0.14^C^	<0.001	0.254
Brainstem	0.25 ± 0.03^A^	0.24 + 0.02^B^	0.22 ± 0.03^C^	<0.001	0.25 ± 0.03^A^	0.24 ± 0.03^B^	0.23 ± 0.03^C^	<0.001	0.013
Orbitofrontal cortex	0.49 ± 0.04^A^	0.46 ± 0.03^B^	0.43 ± 0.05^C^	<0.001	0.51 ± 0.04^A^	0.50 ± 0.05^A^	0.46 ± 0.04^B^	<0.001	<0.001
Right insular/Limbic cluster	0.61 ± 0.04^A^	0.60 ± 0.04^A^	0.57 ± 0.05^B^	<0.001	0.63 ± 0.05^A^	0.62 ± 0.04^A^	0.60 ± 0.05^B^	<0.001	<0.001
Caudate nuclei	0.56 ± 0.06^A^	0.52 ± 0.05^B^	0.48 ± 0.04^C^	<0.001	0.57 ± 0.06^A^	0.53 ± 0.05^B^	0.52 ± 0.06^B^	<0.001	0.003
Hippocampus	0.68 ± 0.04	0.69 ± 0.05	0.67 ± 0.08	0.079	0.68 ± 0.04	0.69 ± 0.04	0.68 ± 0.04	0.222	0.829

Data represented as normalized values (average gray matter volume within each given region of interest) (mean ± SD).

*Results of one-way ANOVA representing age-group differences within each sex.

^†^Results of one-way ANOVA representing between-sex differences.

^A,B,C^Within each row and within each sex group, each letter represents significantly different group means based on Tukey’s *post hoc* testing.

**FIGURE 2:**
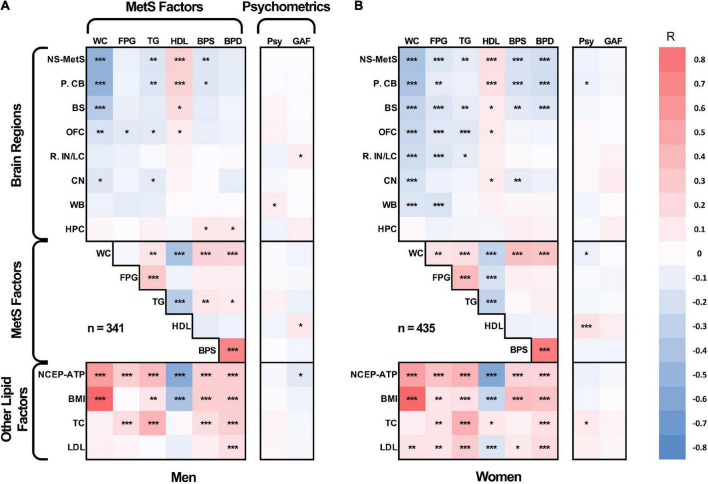
Age-adjusted partial Pearson’s correlation tables in men **(A)** and women **(B)** for gray matter densities of the neural signature of metabolic syndrome (NS-MetS); its substructures: posterior cerebellum (P. CB), brainstem (BS), orbitofrontal cortex (OFC), right insular/limbic cluster (R. IN/LC), caudate nuclei (CN); control brain regions: whole brain (WB) and hippocampus (HPC); components of metabolic syndrome: waist circumference (WC), log-transformed fasting plasma glucose (FPG), log-transformed triglycerides (TG), high-density lipoprotein cholesterol (HDL), systolic blood pressure (BPS), and diastolic blood pressure (BPD); other biometric values: national cholesterol education program adult treatment plan III metabolic syndrome index score (NCEP-ATP), body mass index (BMI), total cholesterol (TC), and low density lipoprotein cholesterol (LPL); and cognitive/psychometric function scores: global assessment of functioning scale (GAF), and latent variable of GOBS psychometric tests composite (Psy). ***Significant at *p* ≤ 0.001, **significant at *p* ≤ 0.01, *significant at *p* < 0.05.

We also created an age-adjusted figure, which incorporates the demographic characteristics from Tables 1, 2 for each sex group using partial correlation with the effect of age regressed out (Figure 2). This figure is helpful for visualizing age-adjusted correlations in our sample cohort between GMV in the NS-MetS brain regions (with the addition of whole brain and hippocampus), correlations between MetS values, and correlations between other lipid measures of interest (NCEP-ATP III scores, body mass index, total cholesterol, and LDL cholesterol). The figure also indicates the strength and significance of each correlation between the NS-MetS regions and MetS factors most relevant for each sex. For example, after adjusting for age, we can appreciate that the relationship between waist circumference (WC) and posterior cerebellar GMV is strong in both men (*r* = –0.50) and women (*r* = –0.40), *p* ≤ 0.001.

Table 3 (men) and 4 (women) represent the results from our linear regression analyses with each table sub-divided by age-group. A total of eight linear regression analyses were conducted for each brain structure within each age-group. This allowed us to identify which of MetS’s five components (WC, FPG, TG, HDL, BP, with the addition of age) most significantly predicted GMV in each region after adjusting for every other covariate.

**TABLE 3:** MetS components’ relationship with gray matter volume in neural signature of metabolic syndrome substructures, whole brain, and hippocampus in men.

	Brain regions	Significant predictors in model (*P-*value)	β^3^	Std. error	Model summary *R*^2^	Model *P*-value
Early adult men (*n* = 137, 20.8 ± 2.2 years, range = 18–25)	NS of MetS	WC (<0.001)** TG (0.005)*	WC (–0.002) TG (–0.031)	WC (0.000) TG (0.011)	0.372	<0.001*
	P. Cerebellum	WC (<0.001)** TG (0.005)*	WC (–0.004) TG (–0.069)	WC (0.001) TG (0.024)	0.367	<0.001*
	Brainstem	WC (<0.001)**	WC (–0.001)	WC (0.000)	0.165	<0.001*
	OFC	*n.s.*	*n.s.*	*n.s.*	0.063	0.024
	R. Insular/Limbic	TG (0.004)*	TG (–0.024)	TG (0.008)	0.050	0.048
	Caudate	TG (0.016)	TG (–0.029)	TG (0.012)	0.061	0.027
	Whole Brain	Age (<0.001)** TG (0.001)* HDL (0.018)	Age (–0.003) TG (–0.009) HDL (0.000)	Age (0.001) TG (0.003) HDL (0.000)	0.231	<0.001*
	Hippocampus	*n.s.*	*n.s.*	*n.s.*	–0.013	0.650
Young adult men (*n* = 137, 34.8 ± 6.1 years, range = 26–45)	NS of MetS	WC (<0.001)**	WC (–0.002)	WC (0.000)	0.269	<0.001*
	P. Cerebellum	WC (<0.001)**	WC (–0.005)	WC (0.001)	0.242	<0.001*
	Brainstem	WC (0.011) FPG (0.030)	WC (0.000) FPG (–0.017)	WC (0.000) FPG (0.008)	0.188	<0.001*
	OFC	Age (0.016) FPG (0.026)	Age (–0.001) FPG (–0.026)	Age (0.000) FPG (0.011)	0.078	0.010
	R. Insular/Limbic	*n.s.*	*n.s.*	*n.s.*	0.035	0.098
	Caudate	Age (<0.001)**	Age (–0.003)	Age (0.001)	0.136	<0.001*
	Whole Brain	Age (<0.001)**	Age (–0.001)	Age (0.000)	0.154	<0.001*
	Hippocampus	*n.s.*	*n.s.*	*n.s.*	0.000	0.427
Middle-aged men (*n* = 67, 54.9 ± 5.7 years, range = 46–65)	NS of MetS	Age (0.008)* WC (0.027)	Age (–0.004) WC (–0.001)	Age (0.001) WC (0.001)	0.161	0.010
	P. Cerebellum	WC (0.017)	WC (–0.003)	WC (0.001)	0.131	0.024
	Brainstem	Age (0.015)	Age (–0.001)	Age (0.001)	0.072	0.104
	OFC	Age (0.003)*	Age (–0.003)	Age (0.001)	0.169	0.008*
	R. Insular/Limbic	Age (0.009)*	Age (–0.003)	Age (0.001)	0.049	0.173
	Caudate	*n.s.*	*n.s.*	*n.s.*	–0.058	0.875
	Whole Brain	Age (<0.001)**	Age (–0.003)	Age (0.000)	0.385	<0.001*
	Hippocampus	Age (0.010)	Age (–0.003)	Age (0.001)	0.105	0.047

Predictor significance is determined using partial correlation to control for all other variables (waist circumference, fasting plasma glucose, triglycerides, HDL cholesterol, blood pressure, age).

*Significant at *p* ≤ 0.008 after correcting for multiple comparisons, **significant *p* < 0.001, and not significant (*n.s*).

**FIGURE 3:**
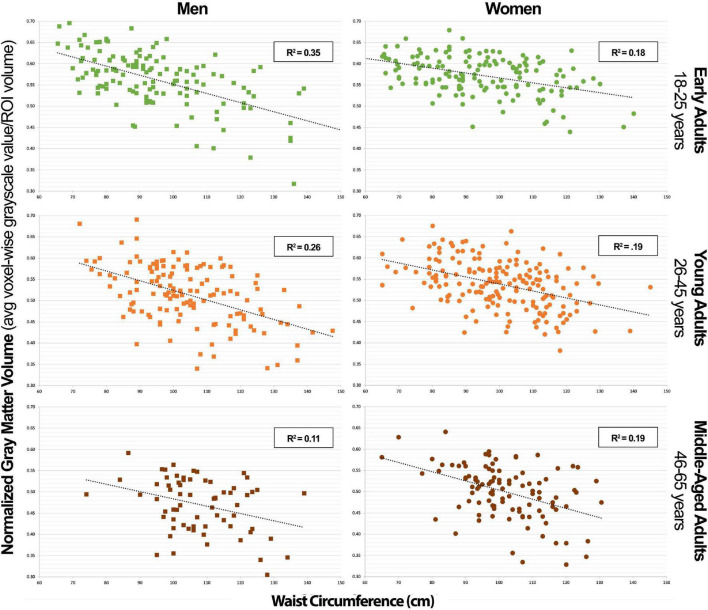
Combined neural signature of metabolic syndrome gray matter volume vs. waist circumference scatterplot series.

**FIGURE 4:**
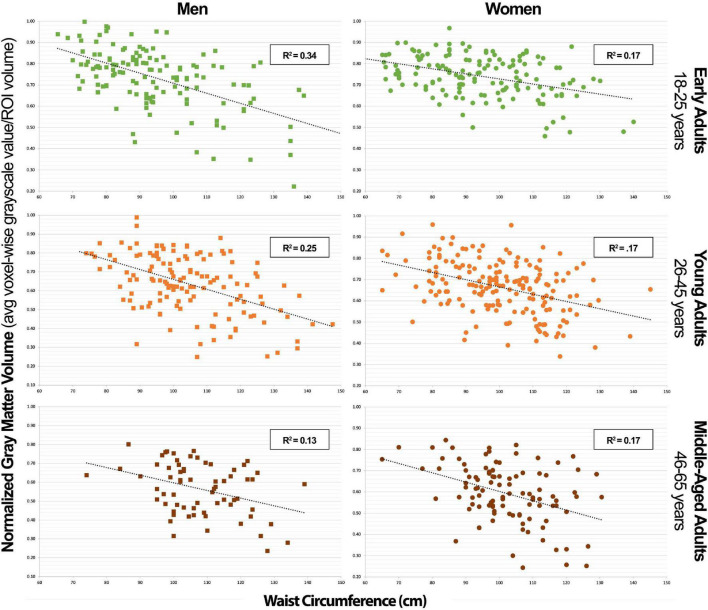
Posterior cerebellar gray matter volume vs. waist circumference scatterplot series.

In Table 3, we can appreciate that the MetS components that most strongly predict GMV in the NS-MetS. In early adult men, these are the combined values of WC and triglycerides (TG), accounting for an effect size of *r*^2^ = 0.372 (also Figure 3). This indicates that 37.2% of the variance in GMV in the NS-MetS can be explained by the combined effects of WC and TG. In young adult men, WC was the single strongest predictor (*r*^2^ = 0.269). In middle-aged men, age and WC were the two strongest predictors (*r*^2^ = 0.161). Overall, WC was the most significant predictor of GMV in the NS-MetS and posterior cerebellum in early, young, and middle-aged adult men (Figure 4). TGs were only seen as significant in early adult men, albeit with small effect sizes (*r*^2^ = 0.050 for the right insular/limbic cluster and *r*^2^ = 0.061 for the caudate). Further, age—within the age-group variance—appeared as the most significant predictor of GMV in the orbitofrontal cortex, caudate, brainstem, right insular/limbic cluster and in the whole brain for young and middle-aged adult men.

In Table 4, we can appreciate that WC was the single strongest predictor of GMV in the NS-MetS in early adult women (*r*^2^ = 0.193) (Figure 3). The combined association of WC and age were the strongest predictors of GMV in the NS-MetS for young adult women (*r*^2^ = 0.254), and WC and FPG were the strongest predictors of GMV in middle-aged women (*r*^2^ = 0.226). Overall, WC was the most significant of the MetS components in predicting GMV in women of all age groups and in almost every region. Our data indicate that each MetS component contributed to GMV in more brain regions in women as compared to men, but the effects of MetS components appeared larger in men than in women, predominantly in the posterior cerebellum. Secondary to WC in women were age, FPG, and TG, with the latter two expressing the largest correlation with GMV in middle-aged women.

**TABLE 4:** MetS components’ relationship with gray matter volume in neural signature of metabolic syndrome substructures, whole brain, and hippocampus in women.

	Brain regions	Significant predictors in model (*P*-value)	β^3^	Std. error	Model summary R^2^	Model *P*-value
**Early adult women** (*n* = 148, 20.7 ± 2.2 years, range = 18–25)	NS of MetS	WC (0.001)*	WC (–0.001)	WC (0.000)	0.193	<0.001*
	P. Cerebellum	WC (0.003)*	WC (–0.002)	WC (0.001)	0.164	<0.001*
	Brainstem	TG (0.006)* Age (0.020)	TG (–0.016) Age (0.002)	TG (0.006) Age (0.001)	0.117	0.001*
	OFC	WC (0.008)* FPG (0.023)	WC (–0.001) FPG (–0.045)	WC (0.000) FPG (0.020)	0.128	<0.001*
	R. Insular/Limbic	WC (<0.001)** Age (0.012)	WC (–0.001) Age (0.004)	WC (0.000) Age (0.002)	0.122	<0.001*
	Caudate	*n.s.*	*n.s.*	*n.s.*	0.019	0.189
	Whole Brain	WC (0.045)	WC (0.000)	WC (0.000)	0.017	0.209
	Hippocampus	*n.s.*	*n.s.*	*n.s.*	–0.013	0.653
**Young adult women** (*n* = 184, 34.7 ± 5.9 years, range = 26–45)	NS of MetS	WC (<0.001)** Age (0.003)*	WC (–0.001) Age (–0.002)	WC (0.000) Age (0.001)	0.254	<0.001*
	P. Cerebellum	WC (< 0.001)** Age (0.010)	WC (–0.003) Age (–0.001)	WC (0.001) Age (0.001)	0.201	<0.001*
	Brainstem	WC (0.001)*	WC (0.000)	WC (0.000)	0.101	<0.001*
	OFC	Age (<0.001)** WC (0.006)*	Age (–0.002) WC (–0.001)	Age (0.001) WC (0.000)	0.139	< 0.001*
	R. Insular/Limbic	WC (0.017)	WC (–0.001)	WC (0.000)	0.049	0.021
	Caudate	Age (0.025)	Age (–0.001)	Age (0.001)	0.056	0.012
	Whole Brain	Age (<0.001)** WC (0.003)* FPG (0.014)	Age (–0.001) WC (0.000) FPG (–0.013)	Age (0.000) WC (0.000) FPG (0.005)	0.230	<0.001*
	Hippocampus	BP-Sys (0.035)	BP-Sys (0.000)	BP-Sys (0.000)	0.009	0.273
**Middle-aged women** (*n* = 103, 53.1 ± 5.3 years, range = 46–65)	NS of MetS	WC (<0.001)** FPG (0.031)	WC (–0.002) FPG (–0.038)	WC (0.000) FPG (0.017)	0.226	<0.001*
	P. Cerebellum	WC (<0.001)**	WC (–0.004)	WC (0.001)	0.173	<0.001*
	Brainstem	FPG (<0.001)** WC (0.002)* Age (0.024)	FPG (–0.021) WC (–0.001) Age (–0.001)	FPG (0.007) WC (0.000) Age (0.000)	0.257	<0.001*
	OFC	TG (0.016) WC (0.024)	TG (–0.022) WC (–0.001)	TG (0.000) WC (0.009)	0.154	0.001*
	R. Insular/Limbic	TG (0.015) HDL (0.028)	TG (–0.030) HDL (–0.001)	TG (0.012) HDL (0.000)	0.112	0.007
	Caudate	*n.s.*	*n.s.*	*n.s.*	0.016	0.278
	Whole Brain	Age (0.020) FPG (0.034)	Age (–0.001) FPG (–0.015)	Age (0.000) FPG (0.007)	0.127	0.004*
	Hippocampus	*n.s.*	*n.s.*	*n.s.*	0.038	0.134

Predictor significance is determined using partial correlation to control for all other variables (waist circumference, fasting plasma glucose, triglycerides, HDL cholesterol, blood pressure, age).

*Significant at *p* ≤ 0.008 after correcting for multiple comparisons, **significant *p* < 0.001, and not significant (*n.s*).

For men and women in general, the primary MetS components exhibiting any degree of significance were WC, TG, age (within age groups), FPG and to a lesser extent, HDL. This indicates that most of the neuroanatomical effects driven by MetS are weighted primarily by these components. Moreover, MetS component’s effects on the whole brain’s gray matter were minimal, with age (within age-groups) emerging as the most significant predictor of whole brain GMV in both men and women within age groups. Importantly, none of the MetS components exerted any noteworthy predictive value on hippocampal gray matter volume.

## Discussion

Our first study found that the greatest differences in gray matter volume (GMV) between individuals with MetS and metabolically healthy controls were observed in the posterior cerebellum, brainstem, orbitofrontal cortex, right insular/limbic cluster (involving the right posterior insula and right amygdala), and caudate nuclei (Figure 1). Other groups have reported similar findings, including an inverse relationship between WC and cerebellar GMV (Kurth et al., 2013) and decreased GMV density of the right insula in individuals with MetS associated with allostatic load (Zsoldos et al., 2018).

The cerebellum has long been known to be involved in motor control and coordination, but recent studies have shown that it also plays an important role in cognition (Schmahmann, 2018; Kotkowski et al., 2020). The cerebellum serves to integrate information from the cortex to produce an output that is coordinated, precise, and accurate on a millisecond timescale in both motor and cognitive domains. While this is obvious in the setting of motor coordination, the behavioral and cognitive aspects are not readily apparent. In fact, these two functions exhibit a clear anatomical partition in the cerebellum with an anterior lobe that modulates motor function and a posterior lobe that modulates behavioral and cognitive function (Stoodley and Schmahmann, 2009). As posited by Jeremy Schmahmann in 1991: “In the same way as the cerebellum regulates the rate, force, rhythm and accuracy of movements, so may it regulate the speed, capacity, consistency and appropriateness of mental or cognitive processes (Schmahmann, 1991).” For this reason, gray matter impairment in the posterior cerebellum might be responsible for observed cognitive impairment in individuals with longstanding MetS and associated comorbidities such as type 2 diabetes mellitus (T2DM) and obesity.

The nodal stress hypothesis might help explain the link between MetS and the caudate nuclei, orbitofrontal cortex, and amygdala in the context of the reward (or appetitive) network. This hypothesis stipulates that regions with high levels of connectivity, which require higher metabolic demand and blood flow, are preferentially susceptible to damage from oxidative stress (Zhou et al., 2012; Crossley et al., 2014). Thus, the over-stimulation—or chronic stimulation of the reward/appetitive network (orbitofrontal cortex, caudate nuclei, insula)—by over-consuming highly palatable food might partially explain our GMV findings. Alternatively, it is possible that genetically-determined predispositions, such as those discussed by Curran et al. (2013) in chromosome 17 associated with leptin signaling, might account for both brain morphology and obesity findings (Winkler et al., 2010). The effects in these regions may thus be components of vulnerability, outcome, or a combination of the two. Nevertheless, a more sophisticated explanation is warranted to explain the pronounced sex differences observed in our study, where we find that certain MetS components appear to correlate more strongly with more regions of the NS-MetS in women than in men, with the notable exception of the posterior cerebellum, which appears to have a stronger correlation with MetS components in men than in women.

Our most surprising finding in this study was the strong negative association between GMV in the posterior cerebellum and WC. We know that in Mexican American populations, elevated waist circumference is independently associated with insulin resistance and risk for developing T2DM (Mamtani et al., 2013). Thus, it could be postulated that the posterior cerebellum is more susceptible to chronically elevated plasma insulin levels. Insulin in the brain plays an important role in modulating synaptic transmission, maintaining neuronal and glial metabolism, and modulating the neuroinflammatory response (Kleinridders et al., 2014). Insulin resistance in the brain might therefore manifest itself as neuronal degeneration detected as decreased GMV. Insulin-like growth factor (IGF) receptors are also heavily expressed in both the cerebellum and the hippocampus (Verdile et al., 2015) and insulin can cross-react with the IGF receptors. This may explain the neuronal degeneration in the cerebellum, but it does not explain why the hippocampus is spared from decreased GMV under the same conditions. Alternatively, the cerebellum is the only brain region, aside from the hypothalamus, with leptin receptors. This has led some investigators to speculate that leptin resistance might also play a role in cerebellar degeneration (Berman et al., 2012), though our current understanding of the mechanistic processes by which this occurs is still in its infancy.

Insulin in the brain modulates synaptic plasticity, specifically of dopamine neurons involved in the reward and appetitive network (Stouffer et al., 2015). In cases of diet-induced obesity, insulin resistance in the hippocampus—a significant brain structure in the pathophysiology of Alzheimer’s dementia (AD), which happens to densely express insulin receptors—has a negative impact on learning and memory. For example, when insulin is elevated, increased glucose metabolism was observed in the appetitive network, prefrontal cortex, insula, and anterior cingulate cortex, but decreased in the hippocampus and cerebellum (Anthony et al., 2006). Findings such as these that identify common structures to those of AD have generated increased interest in identifying hippocampal pathology mechanisms that relate to MetS, type 2 diabetes, obesity and AD. What our research has shown is that when it comes to gray matter atrophy, the pattern observed with the NS-MetS is very distinct from that of mild cognitive impairment (MCI) and AD. Whereas MCI and AD begin demonstrating gray matter loss in the hippocampus and medial temporal lobe structures prior to symptom onset, the same mechanism cannot be suggested to explain why individuals with MetS end up developing cognitive decline. Instead, other brain regions that have not been as extensively studied but are nonetheless important to cognition, namely the posterior cerebellum, should be given more attention in future research.

Our study’s key strength is that it represents a population-based and heterogenous community sample, as opposed to a disease-based sample, allowing us to investigate relationships between normally distributed characteristics. Additionally, the T1-weighted MRI images available for each of the 776 subjects were of exceptionally high quality, as highlighted in the methods, using a research-dedicated 3T scanner and 1-h long, multi-faceted anatomical brain scans. However, our study also had notable limitations. The largest limitation is its cross-sectional and retrospective design. This method limited our ability to obtain biomarkers which would have made our subject stratification more ideal. For example, using hemoglobin A1c instead of fasting plasma glucose as a metric to define blood glucose levels. The cross-sectional nature of our sample allowed us to calculate correlations but prevented us from deducing causal relationships. Furthermore, we were limited by the number of participants in older age-groups that could have yielded valuable information regarding the effects of MetS factors on older individuals who manifest cognitive decline in higher numbers. Finally, were also limited by a lack of data on when each individual first met diagnostic criteria for MetS and related diseases like diabetes and hypertension along with how long they carried the disease burden. This last limitation is the key in understanding why there could be a lower variance in the correlation data between NS-MetS GMV and WC and the posterior cerebellar GMV and WC in older men (Figures 3, 4). For example, it is likely that men who meet the diagnostic criteria for MetS or have increased WC early in life suffer earlier instances of morbidity and mortality, thus experiencing deleterious brain effects earlier. These same individuals might be less likely to be represented in the older cohort who may have developed increased waist circumference and MetS biomarkers later in life after decades of relative health.

In summary, our results provide a detailed breakdown of important brain regions implicated in the pathophysiology of MetS while identifying the sub-components that are most associated with GMV in these regions. The results also highlight the importance of age and sex in determining the effects of MetS on the brain. Future neurocognitive, structural, and functional analyses in future studies involving participants with MetS, obesity, and diabetes can stand to benefit from our findings in their experimental designs. These data can also aid in generating hypotheses that merit further inquiry, such as probing why the observed sex differences exist, specifically in questions where sex differences might play a role in metabolic signaling, such as in fat distribution differences, menopause, and other potential hormonal effects. Anticipated investigations will also be focused at understanding the mechanistic nature of why certain MetS components correlate with GMV, specifically in the posterior cerebellum.

## Conclusion

This study examined the associations between age groups and between the sexes with each of the five components of metabolic syndrome (MetS) and five brain regions comprising the previously defined neural signature of MetS (NS-MetS). We found that waist circumference (WC) was the strongest predictor of decreased posterior cerebellar gray matter volume (GMV), regardless of age or sex. In men, associations between WC and posterior cerebellum were most pronounced in early adults, an effect that appeared to decrease with increasing age. The inverse was true in women, where the association between WC, (along with TG, and FPG) on the brain’s gray matter volume appeared to be slightly weaker in early adult women but increased with increasing age. Between men and women, the potential of MetS components to predict GMV particularly strong in men but confined primarily to the posterior cerebellum. In women, the correlation between MetS components on GMV was more diffuse across most NS-MetS regions.

## Data availability statement

The data that support the findings of this study are available from DG and JB. Restrictions apply to the availability of these data, which were used under license for this study.

## Ethics statement

All procedures performed in studies involving human participants were in accordance with the ethical standards of the institutional committee, NIH, and with the 1964 Helsinki Declaration and its later amendments or comparable ethical standards. Informed consent was obtained from all individual participants involved in this study. The patient data utilized in this research project has been acquired and is currently archived at the Research Imaging Institute as part of the Genetics of Brain Structure and Function (GOBS) study. The GOBS database constitutes a large (*n* = 1911) series of magnetic resonance and genetics data gathered by DG and JB. This project was funded by a series of NIMH grants (MH078111; MH0708143; MH083824). Part of the resource-sharing agreement for these grants is that GOBS data be de-identified and accessible for future studies.

## Author contributions

EK and PF devised the project and its main conceptual ideas with help from RAD. EK wrote the manuscript with support from PF, RAD, CF, and AG. CF was instrumental in the design of the methods involving data curation and image processing with help from MS. LP assisted in the design of statistical methods and analyses used in the study. MW, JB, DG, RD, and PF obtained the individual subject data and MRI scans used in this study. All authors contributed to the article and approved the submitted version.
